# Dengue Fever-Triggered Malignant Hyperthermia

**DOI:** 10.7759/cureus.15121

**Published:** 2021-05-19

**Authors:** Kanakka Hewage Dammika Madhusankha, Harshani Fernando, Sanupa Kumarasiri, Ganesha G Liyanarachchi

**Affiliations:** 1 Emergency Medicine, National Hospital, Kandy, LKA; 2 Internal Medicine, National Hospital of Sri Lanka, Colombo, LKA

**Keywords:** metabolic crisis, dengue fever, rhabdomyolysis, hyperthermia crisis, non-anaesthetic malignant hyperthermia, dantrolene

## Abstract

Malignant hyperthermia (MH) is a genetic skeletal muscle disorder characterized by hypermetabolic crisis usually triggered by anesthetic drugs. Non-anesthesia-triggered or awake MH is rare or under-reported. Other than anesthetic drugs, identified common triggers are exercise, fever, and viral infection. The literature does not report any awake MH cases triggered by dengue fever. We report a possible case of dengue fever-triggered awake MH. The main aim of reporting this case is to raise awareness of possible malignant hyperthermia in dengue patients and of a clinical grading system (CGS) for early diagnosis, as early treatment with dantrolene sodium reduces the mortality rate.

## Introduction

Malignant hyperthermia is a rare genetic disorder denoted by hypermetabolism in skeletal muscle and is usually triggered after susceptible individuals are given volatile anesthetics or depolarizing muscle relaxants [1].

Life-threatening non-anesthetic-triggered malignant hyperthermia (MH) episodes have been identified as “awake” MH. The characteristic clinical findings are the sudden onset of unexplained tachycardia, hyperthermia, tachypnea, skeletal muscle spasms, severe mixed acidosis, and hyperkalemia [2-4].

Awake or non-anesthetic MH episodes are seen in physically fit individuals who have been exposed to excessive heat, vigorous exercise, febrile and flu-like illnesses like influenza A. Of all the triggers, influenza A causes a highly aggressive onset with a rapid increase in body temperature, rhabdomyolysis, and disseminated intravascular coagulation [1-2,4].

A clinical grading system (CGS) can be used to assess the likelihood of MH based on a scoring system. According to this system, a patient with a score of >50 points has malignant hyperthermia [5,6]. The scoring system helps to identify acute crises in patients who have not been previously recognized as malignant hyperthermia-susceptible individuals or in situations where genetic or in vitro contracture testing is not possible.

## Case presentation

A 29-year-old, previously healthy male walked into the outpatient department (OPD) of the National Hospital of Sri Lanka with dengue non-structural protein 1 (NS1) antigen-positive fever of three days duration. Other than the three-day history of fever, he had no other signiﬁcant past medical history except a failed military fitness test because of body temperature 40°C and muscle cramp after running 1 km four years previously. He denied a recent history of strenuous exercise or the use of illicit drugs. Urine or serum drug screen was not performed due to lack of facility.

On admission to the medical ward, he was conscious and rational. His temperature was 37.3°C, his heart rate was 80 bpm, and his blood pressure was 110/60 mmHg. Hematologic analysis revealed full blood count with WBC 4,850 per microliter (4.850 x 10^9^/L), hematocrit 39%, and platelets 180,000 per microliter (180 x 10^9^/L). One hour after being admitted to the medical ward, he developed a high fever with a temperature of 40.2°C which was treated with active cooling with IV cooled normal saline and ice water sponging. Other clinical features detected were diaphoresis, tachycardia with a heart rate of 140 beats/min, tachypnea with a respiratory rate of 40 breaths/min, skeletal muscle spasms, and limb rigidity. Arterial blood gas (ABG) revealed the following: pH - 6.9, PCO2 - 101 mmHg, HCO3 - 8 (mmol/L), BE - (-14), PO2 - 105 mmHg, SpO2 - 95%, and K+ - 6.8. Hyperkalemia was managed by giving intravenous 10 units of soluble insulin and 50% Dextrose infusion, Intravenous 8.4% bicarbonate 50 ml infusion. Intravenous 10% calcium gluconate 10 ml was given to protect cardiomyocytes.

The patient’s clinical condition deteriorated after 10 minutes, and he developed increasing muscle tone with trismus, opisthotonic posture, hyperthermia, severe metabolic and respiratory acidosis despite hyperventilation, refractory hyperkalemia (8.8 meq/l), and hypocalcemia. ABG results were as follows: PH - 7.03, PCO2 - 100 mmHg, HCO3 - low, and lactate - 10. Urine was brown in color and urine for myoglobinuria report received as positive after 4 hours.

Then the patient was emergently intubated after giving IV atracurium 60 mg (A non-depolarizing muscle relaxant). He progressed to asystolic cardiac arrest, which was treated according to the advanced life support (ALS) protocol. The patient’s hyperkalemia was refractory to insulin and dextrose infusion and bicarbonate infusion. Salbutamol was not started due to tachycardia. He expired after two hours of attempted resuscitation.

## Discussion

Literature review

A study conducted on 286 anesthesia-triggered MH cases was used to develop a clinical grading system (CGS) to assess the likelihood of MH. The CGS (Table 1) assesses the likelihood of MH without the use of specialized diagnostic testing such as muscle biopsy. It is based on points given for abnormal signs and laboratory findings observed during an acute hypermetabolic crisis [7].

**Table 1:** Clinical grading scale for malignant hyperthermia

Clinical Finding (Maximum Score)	Manifestations
Respiratory acidosis (15)	End-tidal CO_2_ >55 mmHg, Partial Pressure Arterial CO_2_ >60 mmHg
Cardiac involvement (3)	Unexplained sinus tachycardia
Metabolic acidosis (10)	Base deficit >8 mEq/L, pH <7.25
Muscle rigidity (15)	Generalized rigidity, severe masseter muscle spasm
Muscle breakdown (15)	Serum creatine kinase >20,000/L units, cola-colored urine, Myoglobinuria, plasma [K^+^] >6 mEq/L
Temperature increase (15)	Rapidly raising temperature, T >38.8°C
Other	Rapid reversal of MH signs with dantrolene sodium (score = 5), elevated resting serum creatine kinase concentration (score = 10)
Family history (15)	Consistent with autosomal dominant inheritance

Another study conducted on 136 non-anesthesia-triggered MH cases found that 75% of patients presenting with post-viral chronic fatigue had a positive caffeine halothane contracture test (CHCT). The author suggests that patients with post-viral chronic fatigue may test positive to the CHCT. This finding requires further study of disordered calcium homeostasis in the skeletal muscle following post-viral fatigability [8,9].

In other reported cases and case series of awake MH, the triggers were sports activity, exercise, fever, and influenza A [3]. MH triggered by dengue fever has not been reported.

Our patient with dengue fever walked to the hospital with stable vital parameters but, shortly after arrival, started showing early features of MH. The main clinical features were unexpected elevation of PCO2 despite increased minute ventilation, skeletal muscle rigidity, high body temperature, tachycardia, severe mixed acidosis, and hyperkalemia. The patient responded poorly to general medical management. He scored 71 points on the MH clinical scoring system, while the cut-off for MH is 50 points (Table 2). Clinically malignant hyperthermia was almost certain [8-9]. As there was no other apparent triggering factor for MH, our patient was diagnosed as a case of awake or non-anesthetic MH triggered by dengue fever.

**Table 2:** Calculation of patient's likelihood of having malignant hyperthermia

Expected Clinical Feature	Patient’s Clinical Finding	Score
Process I – Muscle rigidity	Generalized rigidity	15
Process II – Myonecrosis	Serum creatine kinase and urine myoglobin not available; K^+^ >6	3
Process III – Respiratory Acidosis	PaCO_2 _ >60 mmgh	15
Process IV – Temperature increase	Rapid increase	15
Process V – Cardiac Involvement	Inappropriate tachycardia	3
Others (1)	Base excess more negative than 8 meq/L	10
Other (2)	Arterial pH < 7.25	10
Total score	-	71

The in vitro muscle contracture test (IVCT) and the caffeine halothane contracture test (CHCT) are considered to be the standard tests to identify susceptibility to MH [9]. The clinical grading system is helpful for making a clinical diagnosis in an acute situation where susceptibility to MH has not been evaluated. High clinical suspiciousness using the clinical grading system may help the clinician to start dantrolene sodium early, which reduces the mortality rate if started within 30 minutes.

## Conclusions

Dengue fever can trigger malignant hyperthermia in MH-susceptible patients. Post-trigger clinical findings are similar in anesthetic and non-anesthetic MH. Recognizing hypermetabolic crisis at the early stage is challenging; the key clinical features are unexplained tachycardia, hyperthermia, muscle rigidity, mixed acidosis, and rhabdomyolysis. The use of a clinical grading scale (CGS) may help early diagnosis, allowing the early initiation of effective treatment. Dantrolene sodium, a post-synaptic muscle relaxant that reduces contractility and energy expenditure, is an effective treatment.
